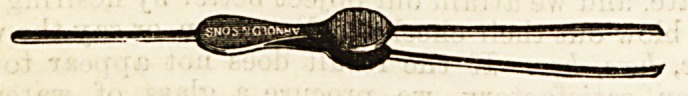# On the Treatment of Otorrhœa in the Aural Department. III

**Published:** 1893-10-28

**Authors:** L. H. Pegler


### The Hospital Clinic.

[*The Editor will be glad to receive offers of co-operation and contributions from members of the profession. All letters should be addressed to* The Editor, The Lodge, Porchester Square, London, W.]

### ST. MARY'S HOSPITAL, LONDON.

### On the Treatment of Otorrhœa in the Aural Department.—III.

The *dry method* of Bezold, which we shall next briefly describe, has undoubtedly proved of signal service in otorrhœa of obstinate character, which has resisted treatment by the wet. It consists in the insufflation of an impalpable powder into the meatus; and practically it may be said that every other drug that has been tried for the purpose has given place to boric acid, minutely pulverised, because it never binds or cakes, and can therefore be syringed away with the greatest ease, or washed out by the discharge so long as the latter persists. A case is selected in which the perforation is large. After first syringing out the meatus and tympanic cavity, and completely removing all purulent material, the parts are thoroughly dried with absorbent wool, and for this purpose some kind of

Cotton Holder.

cotton-holder must be requisitioned. Numerous forms are supplied by the instrument makers, but there is a knack of making a simple rod of copper or steel, such as that figured in the drawing, serve very well. After a little practice it will be found quite easy to wrap a small piece of cotton wool around a perfectly smooth surface. Twists and screws add to the cost of the instrument, and only render it more difficult to remove the sodden wool, which should easily slip off the end and thus save time and trouble, besides preventing the fingers from being soiled. No pain need be inflicted if the end be well protected, and the holder—a thickish being safer than a thin one—inserted with gentleness and caution. To students this simple operation is valuable, by helping to give them something of the much-needed *tactus*, teaching them to gauge by the latter the depth of the meatus in abnormal states, and preparing the way for the acquirement of facility in the performance of more difficult manipulations.

When every trace of moisture has been removed nearly as much powder is sifted into the auditory canal

Form of Politzer Bag Used.

as the latter will hold, and retained there by a small plug of cotton wool, the head being inclined to the opposite side whilst this is being done. In obstinate cases the procedure must be repeated several times a day, until, in fact, the powder ceases to be saturated with discharge and remains quite dry. It is thus not well suited for hospital out-patient practice, since it requires constant and skilled supervision, and absorbs more time than it is possible to give to it in these institutions; without doubt, however, whatever trouble is expended upon it will be amply repaid, for in favourable cases a dry healthy surface is soon established in place of the moist secreting one; but, of course, granulations must be reduced before it can be successfully applied. Mention was made in our first paper of the use of Politzer's bag as a curative agent in the treatment of acute suppuration, but it is scarcely of less importance when the disease has assumed the chronic form. It is rare to meet with a patient having a perforation of long standing who does not tell you he hears better after you have employed it for him. It seems advisable therefore to give a few instructions for the performance of Politzeration, especially because the attendant upon a case may often be required to do it in the surgeon's absence. There are several forms of both air-ball and nozzle, but which of these we select is not of much moment. Mr. Field prefers the nasal pad of the late Peter Allen, of St. Mary's Hospital, which has an aperture for adaptation to each nostril, but a simpler contrivance finds more favour with aurists now-a-days, in which the tube after leaving the air-ball, terminates in a soft teat-like arrangement, made of indiarubber, resembling the mouthpiece of a baby's bottle. This is inserted into one nostril, and retained there by pressure with the fingers and thumb of the operator's left hand. One's object now is to open the nasopharyngeal extremity of the Eustachian tube. In the case of children, it is only necessary to ask them their names, and squeeze the balloon when they are speaking; but in adults, this end of the tube is probably less patent in the normal state, and we attain our object better by desiring them to blow out their cheeks to distension, or say the words *hic, hœc, hoc*. If the result does not appear to have been satisfactory, we procure a glass of water and direct the patient to take a small quantity in his mouth and swallow at a given signal; as he does this, we watch for the upward movement of the thyroid prominence in the middle line of the neck and compress the bag simultaneously; by this means we are enabled to propel air at precisely the right moment through the Eustachian tube into the tympanum. The peculiar and familiar thud which is experienced when the drums are intact, cannot of course be expected to be heard when they are perforated.

There is a strong impression prevailing at St. Mary's, as in all other schools, that the condition of the nasopharynx, in suppurative disease of the ear is of the first importance; and it is therefore the practice to get the mucous membrane of that region into as healthy a state as possible, and keep it so. Postnasal catarrhs are subdued by spraying, syringing, or sniffing up the following alkaline detergent into the nose: ℞. sodæ bicarb gr. xv., sodæbibor gr. xv., acid carbolic gr. iv., aquæ ad ℥ij. This, variously modified by different prescribers, is known as Dobell's solution, and is the *nebula alkalina* of the hospital pharmacopæias. The patient is directed to breathe only through his mouth, as this precaution secures an elevation of the soft palate until it forms a sort of floor to the nasopharynx, then the solution being directed up one nostril, is made after a little practice to return by the other, cleansing and washing out the nasal cavities and nasopharyngeal space in its passage through them. Obstructions to free breathing through the nose are dealt with in the usual way, and post-nasal growths, as well as enlarged tonsils—which in all cases are a great hindrance to the cure of aural disease, through interference with proper drainage and *asepsis*—are removed as a preliminary measure. *Constitutional* treatment of chronic aural suppuration must go hand in hand with local. Cod liver oil and iron tonics often hasten the cure considerably in anæmic and strumous individuals, especially children, whereas in adults we administer iron and quinine or strychnine; the latter remedy is particularly indicated with a view to bracing up the auditory nerve and getting it into as healthy a state as possible, especially where there is evidence of deficiency of nerve tone.

It is regrettable to have to admit that in spite of the various means severally indicated in this article, it is our frequent experience that suppuration associated with perforation of Schrapnell's membrane is remarkably intractable. The ordinary methods of cleansing already detailed are not of much avail in this instance, and a soft rubber intra-tympanic syringe must be employed by which to wash out as thoroughly as possible Prussak's space, with a weak antiseptic solution, the tympanic end of the instrument being carefully insinuated with the aid of a speculum into the perforation. It is only after this has been done, and by the performance of certain operative procedures upon the ossicular chain and space, which need not here be detailed, that much impression can be made upon the diseased parts, and even then the result is by no means always satisfactory.

Having done all we can towards bringing a diseased ear-drum into as satisfactory a condition as possible, it only remains for us to see whether anything further can be tried for the improvement of the hearing by the use of an artificial drum-head. With reference to the employment of this often useful adjuvant, space will only allow of our stating briefly a few facts which are of common experience at St. Mary's: We have at present no definite rules to guide us by which we can give an opinion as to whether an individual patient will benefit by the wearing of an artificial drum-head or not, nor the special form which, of all the numerous ones in vogue, will be most likely to prove successful in each case. The precise position which it is to occupy must be left to the patient himself, after we have shown him how to insert it with the forceps provided for the purpose. As we have no exact knowledge as to its *modus operandi*, beyond the self-evident fact that it assists in communicating the vibrations of the atmosphere to the labyrinth, it is not a matter of surprise that we can assist him so little in the operation, but it may be regarded as fairly conclusive that the substitute should be conveyed along the meatus with the forceps till it rests in contact with the handle of the malleus, or whatever of that ossicle remains, its precise adjustment being effected with the probe end of the instrument, and being solely a matter of individual experience. Every kind of artificial drum membrane must be removed at night; and with regard to the question which one to select, it may be affirmed that nearly as good results are obtained with a simple pellet of moistened (antiseptic) wool, squeezed as flat as a wafer between the finger and thumb as with more elaborate contrivances. These little discs should not be more than a third of an inch in diameter, and the thinner they are the better. Costing nothing, they have a manifest advantage for poor patients. Unfortunately they are a little apt to be forgotten and left in the ear, either entire or in part, thereby creating irritation and a renewal of the suppuration; in fact, like every other form of artificial membrane, they may do so when removed regularly and completely. If this is the case, they must be discontinued, until the mischief has subsided, and the irritated mucous membrane has become more tolerant of their presence.

It should be understood, however, that the artificial membrana tympani invented by Mr. Field and Mr. Ward Cousins, respectively, are also employed at St. Mary's in those fortunate cases that derive special benefit from this form of treatment. L. H. Pegler, M.D.

## Figures and Tables

**Figure f1:**
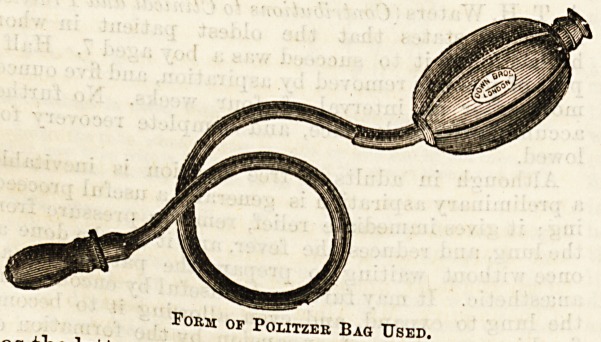


**Figure f2:**